# Calcium dynamics and chromatin remodelling underlie heterogeneity in prolactin transcription

**DOI:** 10.1530/JME-20-0223

**Published:** 2020-10-20

**Authors:** Claire V Harper, Anne V McNamara, David G Spiller, Jayne C Charnock, Michael R H White, Julian R E Davis

**Affiliations:** 1 Department of Biology, Edge Hill University, Ormskirk, Lancashire, UK; 2 Systems Microscopy Centre, Faculty of Biology, Medicine and Health, University of Manchester, Manchester, UK; 3 Endocrine Sciences Research Group, Faculty of Biology, Medicine and Health, University of Manchester, Manchester, UK

**Keywords:** prolactin, transcription, calcium, chromatin, heterogeneity, single cell

## Abstract

Pituitary cells have been reported to show spontaneous calcium oscillations and dynamic transcription cycles. To study both processes in the same living cell in real time, we used rat pituitary GH3 cells stably expressing human prolactin-luciferase or prolactin-EGFP reporter gene constructs loaded with a fluorescent calcium indicator and measured activity using single-cell time-lapse microscopy. We observed heterogeneity between clonal cells in the calcium activity and prolactin transcription in unstimulated conditions. There was a significant correlation between cells displaying spontaneous calcium spikes and cells showing spontaneous bursts in prolactin expression. Notably, cells showing no basal calcium activity showed low prolactin expression but elicited a significantly greater transcriptional response to BayK8644 compared to cells showing basal calcium activity. This suggested the presence of two subsets of cells within the population at any one time. Fluorescence-activated cell sorting was used to sort cells into two populations based on the expression level of prolactin-EGFP however, the bimodal pattern of expression was restored within 26 h. Chromatin immunoprecipitation showed that these sorted populations were distinct due to the extent of histone acetylation. We suggest that maintenance of a heterogeneous bimodal population is a fundamental characteristic of this cell type and that calcium activation and histone acetylation, at least in part, drive prolactin transcriptional competence.

## Introduction

It is widely reported the that transcription of genes is not a static process and can occur in rapid bursts (with second-minute timescales (Ozbudak *et al*. 2002, Blake *et al*. 2003, Raser & O’Shea 2004, Golding *et al*. 2005, Raj *et al*. 2006, Yu *et al*. 2006, Harper *et al.* 2011, Fujita *et al*. 2016)) or longer cycles (with minute–hour timescales (Wijgerde *et al*. 1995, Zenklusen *et al*. 2008, Degenhardt *et al*. 2009, Harper *et al.* 2011, Suter *et al*. 2011, Molina *et al*. 2013)). In eukaryotic cells this has been studied at the population biochemical level using chromatin immunoprecipitation to measure binding of transcription factors to gene promoters (Metivier *et al*. 2003, Kangaspeska *et al*. 2008) or in living single cells using reporter constructs or direct RNA measurements visualise the kinetics of transcription (White *et al*. 1995, Chubb *et al*. 2006, Harper *et al.* 2011, Suter *et al.* 2011, Molina *et al.* 2013, Fritzsch *et al*. 2018).

Transcription of the hormone prolactin (PRL) has been widely shown to be unstable and pulsatile. The presence and timing of pulses is heterogeneous between cells in both primary pituitary cells (Shorte *et al*. 2002, Semprini *et al.* 2009, Harper *et al.* 2010) and clonal pituitary cell lines (Castano *et al*. 1996, Takasuka *et al*. 1998, Semprini *et al.* 2009, Harper *et al.* 2010, 2011). We have shown that activation of the human prolactin promoter occurs in long (~11 h) cycles and we calculated the duration of defined transcriptional ‘on’, ‘off’ and ‘refractory’ periods within this cycle in transcriptionally active cells (Harper *et al.* 2011). Histone acetylation was shown to be involved in generating these cycles. This supports other studies that suggest that transcription bursts/cycles can be regulated by defined periods of histone modification (Blake *et al.* 2003, Metivier *et al.* 2003, Raser & O’Shea 2004, Metivier *et al*. 2006, Raj *et al.* 2006, Kangaspeska *et al.* 2008).

As well as the role of chromatin modifications on transcriptional heterogeneity, the link between calcium signalling and transcription has been well-reported. Studies from around three decades ago showed that calcium was required for the transcription of prolactin (White *et al*. 1981, Day & Maurer 1990, Hoggard *et al*. 1991). This was followed by pioneering work showing that calcium dynamics are related to downstream transcription (Dolmetsch *et al*. 1998, Clapham 2007). Primary pituitary cells and pituitary-derived cell lines have been widely shown to exhibit spontaneous oscillations or spikes in intracellular calcium concentration ([Ca^2+^]_i_) (Schlegel *et al*. 1987, Lewis *et al*. 1988, Wagner *et al*. 1993, Villalobos *et al*. 1998, Shorte *et al*. 2000, Zimber & Simasko 2000, Langouche *et al*. 2001, Van Goor *et al*. 2001, Romano *et al*. 2017) and a relationship has been reported between the presence of calcium spikes and prolactin secretion (Law *et al*. 1989, Charles *et al*. 1999, Van Goor *et al.* 2001). An initial link between calcium spikes and prolactin transcription was suggested (Villalobos *et al*. 2002) but is still not completely understood.

In this study, we focus on two factors that may contribute to the transcriptional heterogeneity of prolactin seen within populations of pituitary cells; calcium dynamics and histone modification.

## Materials and methods

### Materials

Fetal calf serum (FCS) was from Harlan Sera-Lab, Crawley Down, UK, Luciferin was from Bio-Synth, Switzerland. BayK-8644, phenyl methyl sulphonyl fluoride (PMSF) and mammalian protease inhibitor cocktail were from Sigma. Calcium indicator Fluo-4 and Calcium Orange-AM were from Invitrogen.

### Production of stable cell lines and cell culture

Clonal rat pituitary GH3 cells stably transfected with a 5 kb hPRL-luciferase reporter construct (GH3/prolactin-luc cells) or both the 5 kb hPRL-luciferase and 5 kb hPRL-destabilised enhanced green fluorescent protein (d2EGFP) reporter constructs (GH3-DP1) were used as described previously (Takasuka *et al.* 1998, Harper *et al.* 2011). Cells were cultured in DMEM containing 10% v/v FCS and maintained at 37°C 5% CO_2_. Cells were maintained in antibiotic to avoid the loss of transgenes.

### Fluorescence and luminescence imaging

GH3/prolactin-luc cells were seeded in 35-mm glass coverslip-based dishes (IWAKI, Japan) 20 h prior to imaging. Luciferin (1 mM) was added at least 10 h before the start of the experiment, and the cells were transferred to the stage of a Zeiss Axiovert 200 equipped with an XL incubator (maintained at 37°C, 5% CO_2_, in humid conditions) maintained within a darkened room. Cells were loaded with Fluo-4 for 30 min and then time-series imaging was performed using a Fluar x20, 0.75 NA (Zeiss) air objective, with an argon-ion laser at 488 nm. Emitted light was captured through a 505–550 nm bandpass filter from a 545 nm dichroic mirror. Calcium recordings were captured every 1 s for at least 250 s unless stated otherwise. Data were captured using LSM510 software with consecutive autofocus. The microscope and all light emitting devices were then shut down and luminescence images were captured using a photon-counting charge coupled device camera (Orca II ER, Hamamatsu Photonics, UK). Sequential images, integrated over 30 min, were taken using 4 × 4 binning and acquired using Kinetic Imaging software AQM6 (Andor, UK). Bright field images were taken before and after luminescence imaging to allow localization of cells. In the relevant experiments, 0.5 µM BayK8644 was added to the dish at around 100 s during the calcium imaging period.

### Analysis of imaging data

Analysis was carried out using Kinetic Imaging AQM6 software (Andor, UK). Regions of interest were drawn around each single cell, and mean intensity data were collected for both the fluorescence and luminescence time-series. The average instrument dark count (corrected for the number of pixels being used) was subtracted from the luminescence signal.

Assessment for criteria of luminescence activity was determined as follows. In unstimulated experiments, the luminescence values from each cell were normalised to the population average. A cell that maintained normalised luminescence values lower than the average (1-fold) was termed ‘Low’. A cell that maintained normalised luminescence values higher than the average (1-fold) or where the normalised luminescence values varied across the average during the experiment was termed ‘High’. In experiments where the cells were stimulated with 0.5 µM BayK8644, the luminescence values from each cell were normalised to the average of the first two data points for that particular cell. A response to stimulus (transcription rise) was recorded if the data points for that particular cell increased within 3 h and reached a 1.5-fold increase within 4 h. Data is presented as mean ± s.d. and Mann–Whitney non-parametric tests are used using GraphPad Prism. Classification of active or inactive calcium was assessed manually, where active calcium referred to cells showing calcium spikes within the 250 s imaging period. Traces were scored blind. Outlying data points were not excluded from the plots.

### Flow cytometry and fluorescence activated cell sorting (FACS)

GH3-DP1 cells were trypsinised and resuspended in PBS at a concentration of 10^6^ cells/mL, before analysisby flow cytometry using a Coulter-Epics Altra flow cytometer.10,000 cells/sample were analysed. Cells were sorted for low and high expression of prolactin-d2EGFP using FACS with WT GH3 cells used to detect autofluorescence levels. A sample of sorted low and high cells were plated into non-adherent dishes and analysed again after 26 h. For ChIP experiments, at least 1.5 x 10^6^ cells were collected for each of the low, high, unsorted and IgG (unsorted) samples.

### ChIP assays and RT-PCR

Experiments using FACS sorted GH3-DP1 cells (1.5 x 10^6^ per sample) were carried out immediately with the cells in suspension. Formaldehyde was added to each tube at the final concentration of 1% v/v and incubated at room temperature for 15 min. Tubes were kept on ice and then samples were washed twice by centrifugation with PBS supplemented with protease inhibitors (1 mM PMSF and 1x mammalian protease inhibitor cocktail). Cells were resuspended into 500 μL of PBS with protease inhibitors, centrifuged (4 min, 500 ***g*** at 4°C) and the pellet resuspended in 200 μL SDS lysis buffer as described previously (Ashall *et al.* 2009) based on the protocol by Upstate Biotechnology. Immunoprecipitation was carried out using 5 µg of either anti-acetylated H3 (Merck Millipore #06-599), anti-IgG (Merck Millipore #12-370) or anti-Pit-1 (Santa Cruz #X-7) antibody. DNA was extracted and amplified by PCR as described previously (Ashall *et al.* 2009). The primer sequences used were: prolactin Promoter1 left GCAATCTTGAGGAAGAAACTTGA, right AGGCATTCGTTTCCCTTTTC amplifying 347bp of DNA; prolactin Promoter2 left GCATGGGAACTTTAGCATCA, right ATAGCCCCACATTTCCTGTG amplifying 351bp; prolactin Promoter3 left CCTGTGCACATGGACAGAAT, right CCATAGTGGAAGCATTTGGAA amplifying 358bp. PCR products were resolved using agarose gel electrophoresis and densitometry was performed using AQM Advance 6.0 software (Kinetic Imaging, UK). Values were normalised to the unstimulated sample.

## Results

### Temporal variation in basal prolactin transcription and calcium patterns in GH3 cells

Pulses in prolactin transcription have been reported for many years (Takasuka *et al.* 1998, Shorte *et al.* 2002, Semprini *et al.* 2009, Harper *et al.* 2010, 2011, Featherstone *et al.* 2016). In previous work using luminescent and fluorescent microscopy of reporter gene constructs we described the evidence of clearly defined prolactin transcription cycles in single cells, occurring approximately every 11–12 h (Harper *et al.* 2011). These cycles are observed in clonal GH3 cells and also in transgenic primary pituitary cells (with a longer cycle of ~15 h) using reporter constructs of varying promoter length (Harper *et al.* 2011).

**Figure 1:**
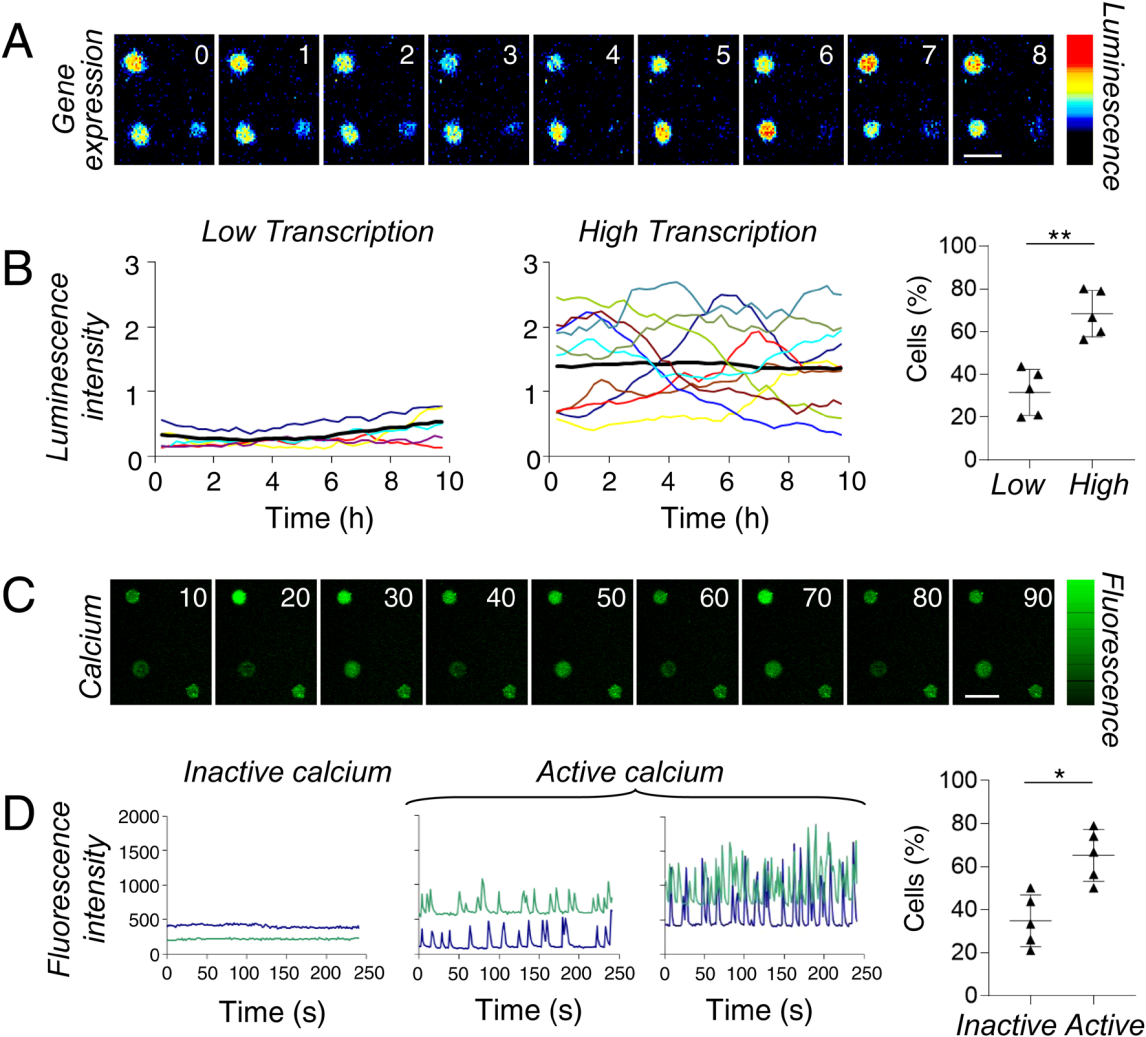
Temporal heterogeneity in prolactin transcription and calcium profiles between pituitary cells. (A and B) GH3 cells stably expressing a 5kb prolactin-luciferase reporter gene (GH3/prolactin-luc cells) show 2 transcription patterns in unstimulated conditions; low and high (see Methods for classification), measured using time-lapse luminescence imaging. Each line represents a single cell, thick black line is experiment average. (C and D) GH3/prolactin-luc cells loaded with Fluo-4 show both inactive and active calcium patterns in unstimulated conditions measured using time-lapse fluorescence imaging. Each line represents a single cell. Scatter plots show the proportion of cells defined by each category in unstimulated conditions where each point represents a single experiment (B and D right panels). Bars in image series represent 50 µm.

Detailed analysis of prolactin transcriptional activity in GH3 cells containing a 5kb prolactin promoter-luciferase reporter gene (GH3/prolactin-luc cells) showed that two transcriptional patterns occurred in unstimulated (basal) conditions: ~35% of cells maintained a relatively even low level of luminescence signal, whereas ~65% showed high or cycling signal over a recorded 10 h period of imaging (Fig. 1A and B; 91 cells, six experiments). This analysis is in agreement with our previous study where ~50% of cells were recorded to show transcription cycles as detected using a binary model of transcription switch times (Harper *et al.* 2011).

GH3 cells, along with other pituitary derived cells, have been widely shown to exhibit spontaneous calcium oscillations (Schlegel *et al.* 1987, Lewis *et al.* 1988, Wagner *et al.* 1993, Villalobos *et al.* 1998, Shorte *et al.* 2000, Zimber & Simasko 2000). Using GH3/prolactin-luc cells loaded with the calcium indicator Fluo-4 to measure changes in intracellular calcium ([Ca^2+^]_i_), we detected spontaneous calcium spikes in around 60% of cells within a 250 s period of imaging (Fig. 1C and D). Patterns varied between cells in the timing of the spikes (Fig. 1D). Approximately 30% of cells showed no calcium spikes within the period of imaging although these cells maintained low basal level of fluorescence above background levels.

### Relationship between calcium dynamics and prolactin transcription profiles in single cells

**Figure 2:**
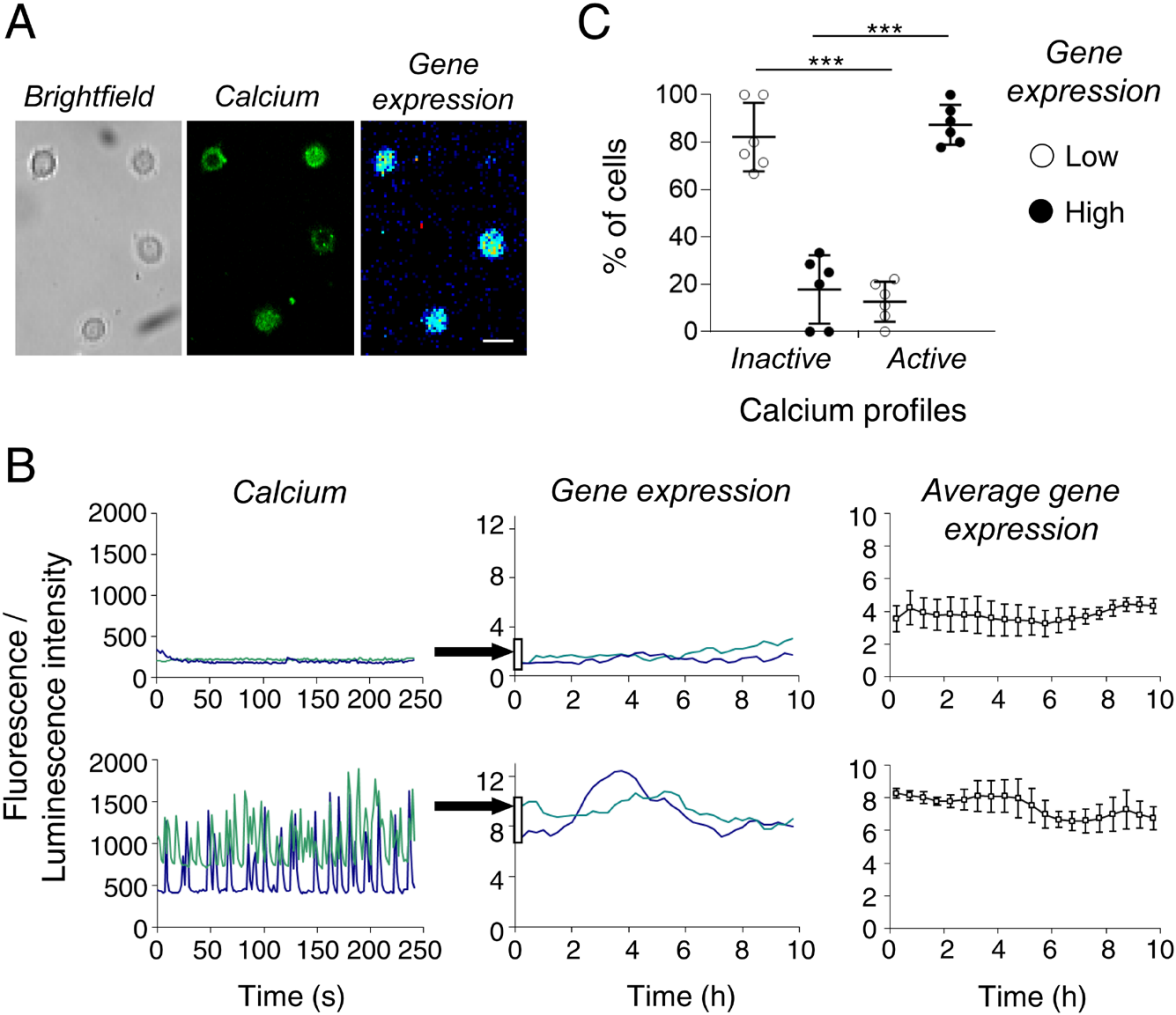
Relationship between calcium patterns and prolactin transcription in pituitary cells in unstimulated conditions. (A and B) Resting calcium profiles and prolactin transcription were measured sequentially in the same cells. (B) Representative cells showing inactive and active calcium patterns and their subsequent transcriptional patterns. Right panels show mean prolactin transcriptional activity from all cells within an experiment that show inactive or active calcium ± s.d.. (C) Scatter plot shows the proportion of cells exhibiting low or high prolactin transcription following active or inactive calcium profiles (six experiments, 91 cells; *P* < 0.01) where each point represents a single experiment. Bar in image represents 20 µm.

A key question that arose from these observations was whether there is a relationship between the basal calcium signal and the basal prolactin expression within a particular cell. To answer this, fields of adherent GH3/prolactin-luc cells were loaded with Fluo-4 to measure [Ca^2+^]_i_ and fluorescent images were captured every 1 s for up to 300 s. Then subsequently, luminescence images were captured on the same field of cells to record prolactin promoter activation (Fig. 2A). [Ca^2+^]_i_ profiles were divided into inactive (those showing no calcium spikes within the period of imaging) or active (those showing any form of calcium oscillations) (Fig. 2B). Luminescence profiles were divided into two categories: low and high as described in Fig. 1. It was clearly apparent that there was a relationship between the [Ca^2+^]_i_ profile and the transcriptional state of the cells (Fig. 2B and C). ~80% of cells showing no calcium spikes showed low maintained levels of prolactin transcription for at least 10 h after the calcium recordings were generated. In cells showing active calcium oscillations, over 80% were displaying high prolactin expression during the following 10 h. This difference was highly significant (Fig. 2C; *P* < 0.001 t-test; six experiments, 91 cells). These data suggest that [Ca^2+^]_i_ may prime a cell for transcriptional activation, or that the [Ca^2+^]_i_ profile determines transcriptional competence of a particular cell.

### Relationship between calcium dynamics and the prolactin transcriptional response to stimulus

Previous research has shown that the transcription of prolactin is cyclical in basal conditions (Harper *et al.* 2011). These cycles are composed of an active ‘on’ phase of transcriptional activation (approximately 4 h) and an inactive ‘off’ phase of transcriptional inactivation (approximately 6.5 h). The ‘off’ phase also contains a refractory period of chromatin modelling (>3 h) where cells cannot respond to stimulus (Harper *et al.* 2011). Therefore the hypothesis is that cells can only respond immediately to stimulus within the non-refractory period of the ‘off’ phase. Application of the acute inducer of [Ca^2+^]_i_ increase, BayK8644, caused a rise in prolactin transcription in 43 ± 3% of cells (*n* = 5 experiments, 77 cells) within the first 3 h following treatment. This supports the above hypothesis, in that not all cells are in a state in which they can be activated immediately. To test whether the transcriptional response to stimulus varied depending on the preceding basal [Ca^2+^]_i_ profile of the cell, GH3/prolactin-luc cells were labelled with fluo-4 and imaged for changes in [Ca^2+^]_i_, during which 0.5 µM BayK8466 was applied to the dish. Prolactin transcription was then measured in the same field of cells for up to 10 h.

**Figure 3:**
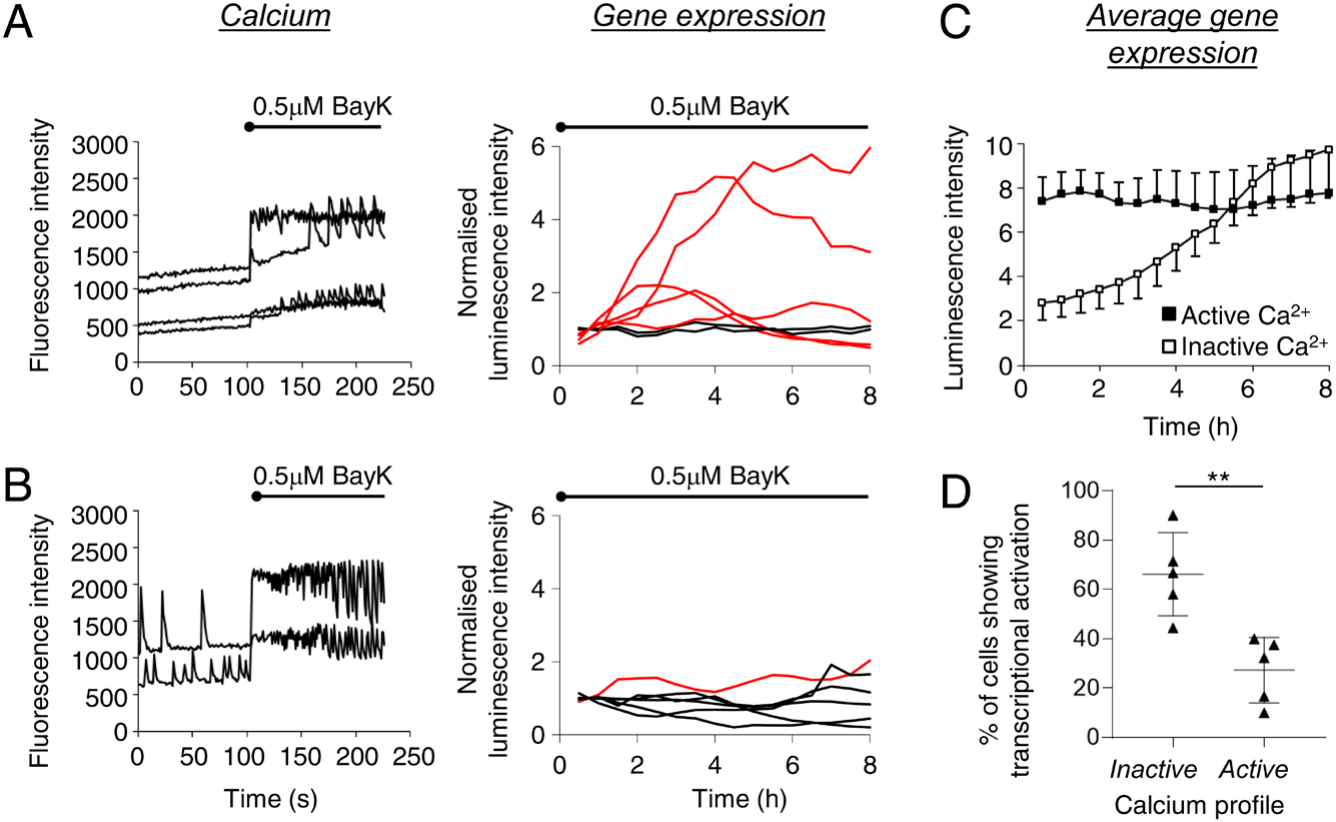
Relationship between calcium patterns and prolactin transcription in pituitary cells in stimulated conditions. (A and B) Calcium profiles and subsequent prolactin transcriptional response patterns following treatment with 0.5 µM BayK8644. The calcium and transcriptional responses to 0.5 µM BayK8644 were measured in cells that showed initial (pre-stimulus) active (A) or inactive (B) resting calcium profiles. Red gene expression traces show a response and black traces show no response to the stimulus (see methods for classification). (C) Mean single cell transcriptional response patterns from cells showing initial active or inactive calcium profiles. Points show mean ± s.d. (D) The proportion of cells showing transcriptional response to stimulus following initial active or inactive calcium profiles, mean ± s.d. (five experiments, 77 cells, *P* < 0.01) where each point represents a single experiment.

Although BayK8644 induced an increase in [Ca^2+^]_i_ in most cells, there was a surprising relationship between the basal [Ca^2+^]_i_ profile of a cell before stimulus and its transcriptional response to the stimulus (Fig. 3). The majority of cells where no basal oscillations in [Ca^2+^]_i_ were recorded prior to stimulus addition responded with a significant transcriptional rise following application of the stimulus (Fig. 3A, C and D; for determination of a significant transcriptional rise see the Materials and methods section). In cells showing basal oscillations in [Ca^2+^]_i_ before addition of the stimulus, few responded with a stimulus-induced transcriptional rise (Fig. 3B, C and D). This difference was highly significant (Fig. 3D; 67 ± % in inactive cells compared to 26 ± 8% in active cells; *P* < 0.01, *t*-test, five experiments, 77 cells).

These data, taken together with those of Fig. 2, suggest that cells showing basal oscillations in [Ca^2+^]_i_ are the prolactin-transcriptionally active population but are less able to respond immediately to acute application of stimulus. In contrast, cells showing no basal [Ca^2+^]_i_ oscillations are transcriptionally dormant (within our experimental detection range) but poised to generate an immediate transcriptional response to the calcium stimulus.

### Temporal heterogeneity in prolactin transcription in clonal GH3 cells

**Figure 4:**
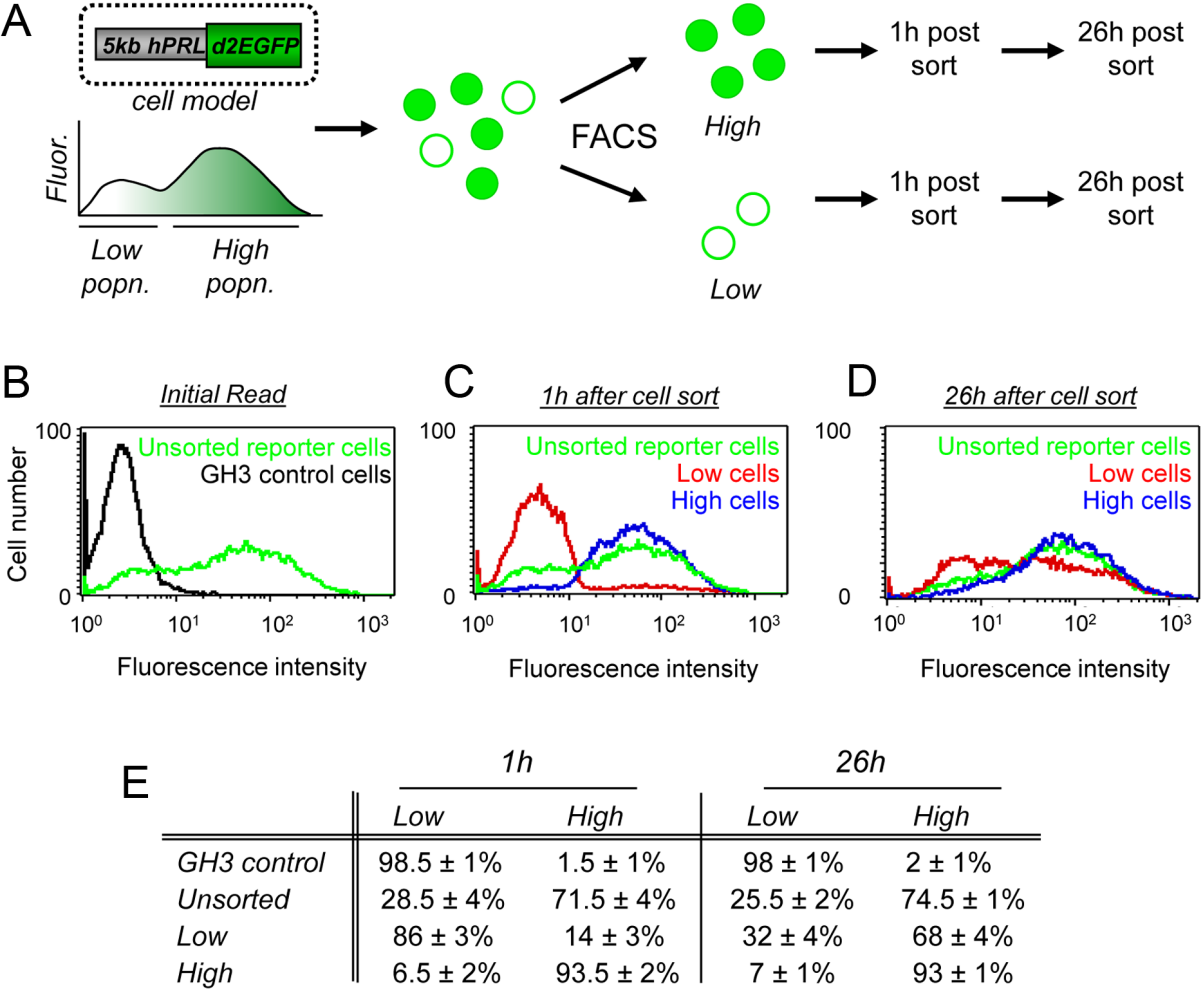
Maintenance of heterogeneity between clonal cells. (A) Model showing protocol. GH3 cells stably expressing a 5 kb prolactin-destabilised EGFP reporter gene (GH3-DP1 cells) were sorted for basal prolactin expression level using FACS. The fluorescence of these sorted cell populations was then measured after 1 h and 26 h. (B) Variation in basal prolactin gene expression in clonal GH3-DP1 cells (green trace) compared to the WT GH3 cell line (black trace). Measurement of fluorescence levels in High (blue trace) and Low (red trace) expressing GH3-DP1 cells following FACS after 1 h (C) and 26 h (D). Data from one representative experiment are shown. (E) Table showing the proportion of cells ± s.d. classified as High or Low prolactin expression 1 h and 26 h post-FACS in GH3 cells (control), unsorted cells, low expressing cell population and high expressing cell population (three experiments).

Several reports using reporter gene constructs have shown that clonal and primary pituitary cells display heterogeneity in the levels of human prolactin expression (Castano *et al.* 1996, Takasuka *et al.* 1998, Shorte *et al.* 2002, Semprini *et al.* 2009, Harper *et al.* 2010, 2011, Featherstone *et al*. 2011). To estimate the extent of basal cellular heterogeneity, GH3 cells stably expressing prolactin-d2EGFP were analysed using flow cytometry (Fig. 4A). WT GH3 cells were used as an auto-fluorescence control. The reporter gene fluorescence intensity within the unstimulated cell population varied over two orders of magnitude indicating cellular variation in the expression of prolactin (Fig. 4B). The distribution of the cell population was bimodal suggesting that there may be two dominant groups of cells, high prolactin expression and low prolactin expression. We have previously shown that cells switch from a transcription ‘on’ state to an ‘off’ state in unstimulated conditions over the duration of several hours (Harper *et al.* 2011) so that these data support that view. Using fluorescence-activated cell sorting, the cells were sorted into two populations; ‘Low’ (~30% of the total population) and ‘High’ (~70% of the total population). The fluorescence levels of these sorted populations were re-analysed after 1 and 26 h to measure the dynamic responsiveness of individual cells (Fig. 4A, C, D and E). After 26 h, the High cell population maintained a similar distribution. But in contrast, the Low population of cells had changed, reverting back into the bimodal distribution shown in the unsorted population (Fig. 4D and E). This clearly shows that the fluorescence expression level of the cells is transient, with cells capable of switching between low and high transcriptional states.

### Relationship between prolactin transcription and histone modification status

**Figure 5:**
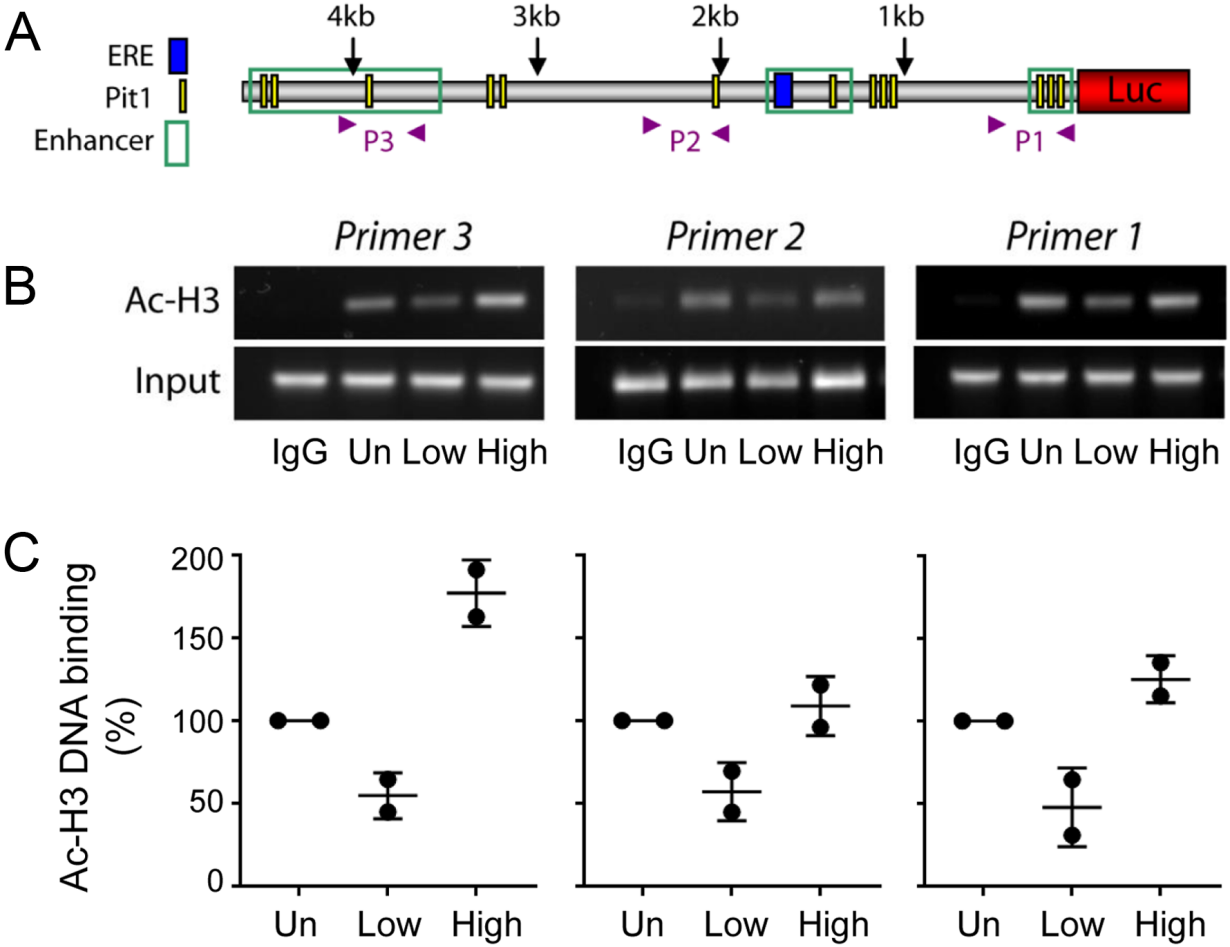
Relationship between level of prolactin transcription and chromatin status at the prolactin promoter. (A) Location of target sites for amplification within the proximal prolactin promoter (designated P1, P2 and P3). GH3-DP1 cells expressing prolactin-eGFP were sorted by level of basal prolactin transcription using FACs (Fig. 4). Cells were classified as unsorted (Un), low transcription (Low) and high transcription (High). (B and C) The level of Acetylated histone H3 was measured using ChIP across the three amplification sites (two experiments, mean ± s.d.).

We have previously suggested that the cycles in prolactin transcription are modulated by histone acetylation, in particular proposing that the refractory period of transcriptional activation may be the result of a period of closed chromatin (Harper *et al.* 2011). To determine in more detail whether the extent of histone acetylation changes during prolactin transcription cycles, GH3-DP1 cells were sorted into populations of Low and High basal prolactin expression by FACS as described above (Fig. 4A). Chromatin immunoprecipitation (ChIP) was immediately performed on these cell populations, using unsorted GH3-DP1 cells as a comparison. Three sites were selected within the human prolactin promoter to measure the extent of acetylated histone H3 (Ac-H3) bound to the DNA (Fig. 5A). The localisation of these sites was based on the prior knowledge that there are enhancer regions within this promoter (Peers *et al*. 1990, Van De Weerdt *et al*. 2000). Primer 1 was in the proximal enhancer region, primer 2 was 2 kb upstream and primer 3 was in the distal enhancer region 4 kb upstream. All three regions contained Pit-1 binding sites, the critical transcription factor for prolactin expression (Fig. 5A). In the Low prolactin transcription cell population (also containing cells in a transcriptional refractory phase (Harper *et al.* 2011)) there was a decrease in Ac-H3 bound to all three sites in the human prolactin promoter when compared to transcriptionally High population of cells (Fig. 5B and C). This implies that the chromatin was more accessible during periods of high prolactin transcription. In contrast, the extent of Pit-1 binding remained consistent across the low and high prolactin transcriptional cell populations (Supplementary Fig. 1, see section on supplementary materials given at the end of this article), suggesting that Pit-1 remains bound to the DNA during cycles of prolactin transcription in unstimulated conditions and that the cycles in transcription are not due to cycles in Pit-1 binding.

## Discussion

**Figure 6:**
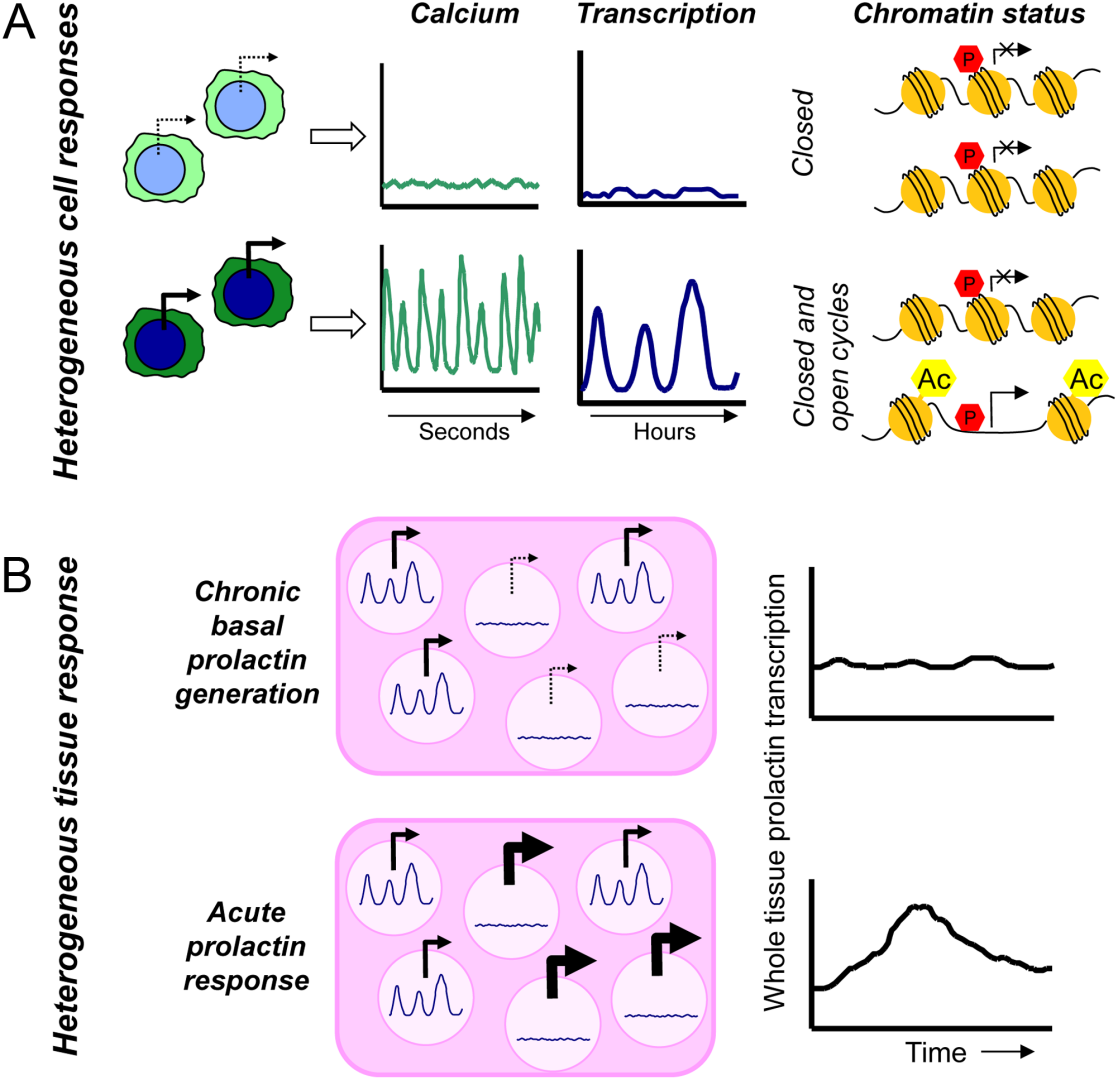
Schematic showing cellular heterogeneity in single pituitary cells and pituitary cells within a tissue. (A) Relationship between calcium profile, prolactin transcription and chromatin status in single pituitary cells. (B, top panel) In basal conditions a subset of cells within pituitary tissue is expressing prolactin at any one time, resulting in low, chronic basal expression of prolactin across the tissue. (B, bottom panel) In stimulated conditions, the cells showing low prolactin transcription within the tissue respond to the stimulus, mounting an acute surge of prolactin expression.

Cycles in prolactin gene expression have been well-reported in the literature (Shorte *et al.* 2002, Semprini *et al.* 2009, Harper *et al.* 2010, 2011, Featherstone *et al.* 2016) but here we add new mechanistic information about how these cycles may occur. We show that within a clonal population of resting GH3 cells there is variability in the extent of prolactin expression, calcium dynamics and histone acetylation. The resting calcium dynamics appear to determine the transcriptional competence of the cell (i.e. whether a cell is transcriptionally active or can respond to a stimulus). Within the population of GH3 cells there were two distinct subpopulations: (1) cells showing inactive calcium, low prolactin transcription and decreased Ac-H3 binding on the human prolactin promoter (closed chromatin) and (2) cells showing active calcium, high or cycling prolactin transcription and increased Ac-H3 binding on the prolactin promoter (open chromatin) (Fig. 6A). In contrast, the levels of Pit-1 binding to the human prolactin promoter were not related to the degree of prolactin transcription implying that Pit-1 may remain bound to the DNA and be controlled by post-translational modifications (Demarco *et al*. 2006).

Work from other groups has suggested a role for calcium signalling in the epigenetic regulation of genes. Sharma and colleagues described a mechanism where increased calcium levels led to changes in chromatin modifications and regulation of gene expression at the level of alternative splicing in cardiomyocytes (Sharma *et al*. 2014) and Raynal *et al.* interestingly showed the potential importance of calcium signalling on the reversal of epigenetic silencing of tumour suppressor genes (Raynal *et al.* 2016). In light of this work, further study should be carried out to determine whether the levels of [Ca^2+^]_i_ set up a cell for transcriptional activation by mechanisms involving chromatin remodelling. Following our earlier work, where we showed that the histone deacetylase inhibitor Trichostatin A affected basal prolactin expression dynamics (Harper *et al.* 2011), it would be interesting to determine whether the relationship between calcium and transcriptional activity can be modulated by disrupting chromatin remodelling.

Maintenance of cellular heterogeneity has been reported to be functionally advantageous at the population level (Paszek *et al*. 2010). We hypothesise that maintenance of a heterogeneous cell population is of innate importance in these hormone producing cells and that the variability in transcription correlated with variability in the calcium status and histone modification status of the cells. Heterogeneity within the cell population was disrupted by separating into two cell populations based on the level of prolactin gene expression. The observation that the low cell population reverted back to having the same transcriptional distribution as the unsorted population within 26 h implies that cells are not constrained to one pattern of expression (high or low), and can switch between states, potentially dependent on the surrounding cells. This observation of maintenance to a steady-state population distribution supports other reports in other clonal cell lines (Sigal *et al*. 2006, Pilbrough *et al*. 2009) although this appears to occur more rapidly in our cells.

The maintenance of a heterogeneous cell population may be important within pituitary tissue, whereby at any fixed time there is a subset of cells expressing prolactin to enable low, chronic basal hormone production (transcriptionally high and cycling cells) but there is also a subset of cells which are ready to mount a response to external stimuli to enable acute hormone production (transcriptionally low cells, Fig. 6B). The observation that an external stimulus (BayK8644) induced prolactin transcription in significantly more calcium inactive cells compared to calcium active cells provided further evidence that there are two cellular sub-populations and supports the idea that it is the inactive cells that are capable of mounting a rapid rise in prolactin transcription. Using similar simultaneous measurements of calcium and rat PRL-luciferase expression in primary rat mammotropes, Villalobos and colleagues (Villalobos *et al.* 2002) showed that the extent of transcriptional response to TSH-releasing hormone was dependent on the resting transcriptional status and the profile of [Ca^2+^]_i_ response. Whether our observations occur in primary rat pituitary cells have not been determined in this study. Heterogeneity in [Ca^2+^]_i_ has also recently been reported in corticotroph cell populations following treatment with the hypothalamic secretagogues corticotrophin-releasing hormone and arginine vasopressin (Romano *et al.* 2017). Our results, together with the findings from these other studies, suggest that cell variability may be mechanistically important at the population level within endocrine tissues, enabling graded responses to varying stimulation levels through changes in cell recruitment. Whether there is a relationship between [Ca^2+^]_i_, prolactin transcription and secretion, namely whether cells with inactive calcium and low transcription are non-secreting, remains to be shown.

In summary we report, for the first time, a significant relationship between the basal calcium dynamics and prolactin transcription in single living GH3 rat pituitary cells. We also show that variability in the extent of histone acetylation on the prolactin promoter determines basal prolactin transcription. It remains to be studied how the heterogeneity within the pituitary cell population is maintained and whether these cells are capable of detecting the status of surrounding cells (through paracrine signalling) and adjusting their role accordingly.

## Supplementary Material

Supplementary Figure 1

## Declaration of interest

The authors declare that there is no conflict of interest that could be perceived as prejudicing the impartiality of the research reported.

## Funding

This work was supported by the Wellcome Trusthttp://dx.doi.org/10.13039/100010269 (Programme Grant #091688 to J R E D and M R H W). C H was funded by The Professor John Glover Postdoctoral Memorial Fellowship.

## Author contribution statement

C V H designed, performed and analysed the research and wrote the manuscript. A V M led the FACS experiments and advised on experiments throughout the study. J C advised on the manuscript. D G S was involved in discussions on the analysis and the manuscript, and managed the Systems Microscopy Centre. M R H W directed the Systems Microscopy Centre and was involved in critical discussions of the work. J R E D advised throughout the study and assisted with writing the manuscript.
