# Supplementary figures and images for: Calcium dynamics and chromatin remodelling underlie heterogeneity in prolactin transcription
Source: J Mol Endocrinol. 2020 Oct 20;66(1):59–69. doi: 10.1530/JME-20-0223 (PMC7774774; doi:10.1530/JME-20-0223)

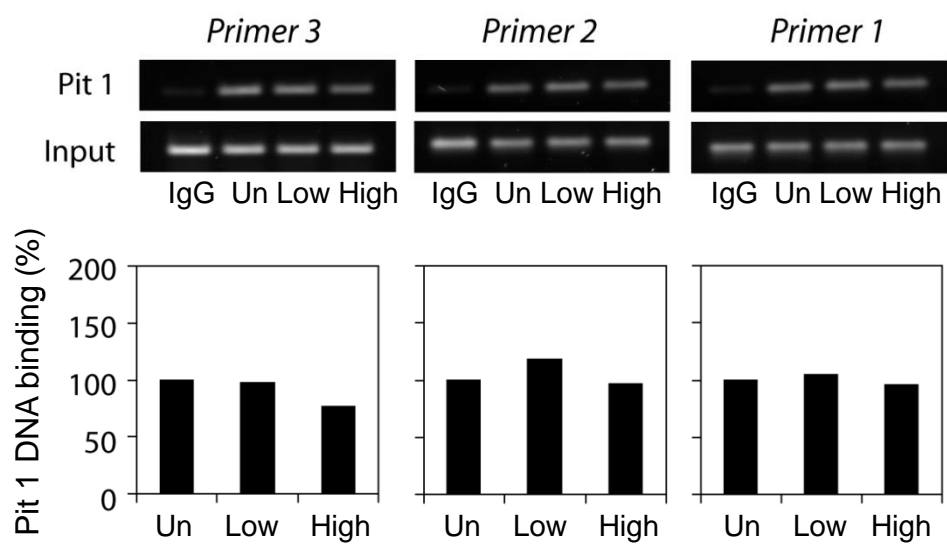

Supplementary Figure 1

Supplement: Supplementary Figure 1 [file supplementary_figure_1.pdf]
